# Effects of timing of umbilical cord clamping for mother and newborn: a narrative review

**DOI:** 10.1007/s00404-023-06990-1

**Published:** 2023-03-29

**Authors:** Juliane Herold, Harald Abele, Joachim Graf

**Affiliations:** 1 Section of Midwifery Science, Institute for Health Sciences, University Hospital Tübingen, Hoppe-Seyler-Str. 9, 72076 Tübingen, Germany; 2 Department for Women’s Health, University Hospital Tübingen, Calwerstr. 7, 72076 Tübingen, Germany

**Keywords:** Umbilical cord clamping, Newborn outcomes, Maternal outcomes, Quality of evidence, Narrative review

## Abstract

**Objective:**

This narrative review was performed to evaluate the correct timing of umbilical cord clamping for term infants. It was intended to determine any advantages or disadvantages from early or delayed cord clamping for newborns, infants or mothers.

**Methods:**

A systematic search on two databases was conducted using the PICO pattern to define a wide search. Out of 43 trials, 12 were included in this review. Three of the included studies are meta-analyses, nine are randomized controlled trials.

**Results:**

Early or delayed cord clamping was defined differently in all the included trials. However, there are many advantages from delayed cord clamping of at least > 60 s for newborns and infants up to 12 months of age. The trials showed no disadvantages for newborns or mothers from delayed cord clamping, except for a lightly increased risk of jaundice or the need for phototherapy.

**Conclusion:**

Delayed umbilical cord clamping for term infants should be performed. Further research is needed to improve knowledge on physiological timing of umbilical cord clamping in term infants, which also leads to the same advantages as delayed cord clamping.

## What does this study add to the clinical work

**Table:** 

This narrative review was performed to evaluate the correct timing of umbilical cord clamping for term infants. The trials showed no disadvantages for newborns or mothers from delayed cord clamping, except for a lightly increased risk of jaundice or the need for phototherapy.	

## Introduction

### Umbilical cord clamping—overview

The correct timing of umbilical cord clamping for term newborns has long been debated in obstetrics [1–3]. This is a usual intervention during the active or passive management of the third stage of labour, and, the question arises as to whether the neonatal outcome after different timings of cord clamping should be investigated. Active management includes the prophylactic administration of uterotonic medication, cord clamping and controlled traction of the umbilical cord to deliver the placenta. Passive management is described as waiting for physiological signs of placental detachment before it is spontaneously delivered. Since 2014, the WHO recommends a waiting period of 1–3 min before cord clamping after the birth of term infants [4].

The maternal outcome of active management has already been thoroughly documented: it decreases the risk of postpartum haemorrhage [5]. Studies have been conducted with regard to the handling of the third stage of labour, in which obstetricians and midwives took part. These studies indicate that 73% of the midwives in the UK prefer active management and 41% usually clamp the umbilical cord within 20 s after the birth of term infants [6].

There is ample evidence showing the advantages for term infants when the cord was clamped at a later point in time, e.g. 60 s after birth [7]. The advantages for term infants include higher haemoglobin levels, a decreased risk of anaemia and lower rates of chronic lung disease [7]. There is also evidence proving the longer term advantages for term infants whose cord was clamped more than 60 s after birth, ranging up to 12 months of life [8].

The actual guideline for obstetrics in Germany recommends waiting at least 1 min and up to 5 min, or when it stops pulsating, before cord clamping [9]. The guideline from paediatrics also recommends delayed cord clamping between 1 to 3 min after birth [10].

### Aims

This narrative review aims to evaluate the timing of umbilical cord clamping for term infants. Furthermore, the review was conducted to expound any advantages and disadvantages from early or delayed cord clamping for mothers, newborn and infants. To improve the evidence-based work of midwives in Germany, the handling of the third stage of labour should be critically evaluated.

## Methods

### Study design

This study design (narrative review) was chosen to detect the actual meta-analyses, systematic reviews and RCTs covering the research question of this review. Furthermore, the study design offers an opportunity to summarize all study results achieved since 2011 and to survey the current state of research. The search strategy adheres to the standards of a systematic search to decrease the risk of selection bias [11].

### Search strategy

The PICO pattern was used to differentiate the search strategy. *Patient*s were pregnant women who gave birth at > 37 weeks of gestational age and their newborns. The *intervention* was declared as the time of umbilical cord clamping. Therefore, the *comparison* refers to the type of intervention to compare the outcome of early or delayed cord clamping management. The *outcome* was defined as measurable short- and long-term effects for the baby. To determine if there were any disadvantages in connection with the cord clamping methods for the mother, whether active or passive management of the third stage of labour was performed was not specified. This led to the central research question: Which timing of umbilical cord clamping on term infants provides advantages for the newborn and produces no disadvantages for mother or newborn?

An electronic search in the Cochrane library and PubMed within a time range from 3^rd^ October to 1^st^ November 2022 was performed. The language for both databases was restricted to German and English. The searched article types were predetermined as meta-analyses, systematic reviews, randomized controlled trials and clinical trials from the last 10 years. A search string for an advanced search was created to extract data to follow the guidelines for systematic search and to improve the reproducibility. The first sequence chosen was “effects umbilical cord clamping” which should be mentioned in the title or abstract. The second sequence was supposed to exclude the literature concerning preterm birth. Search string: (effects umbilical cord clamping) [Title/Abstract]) NOT (preterm [Title])). A filter was added to search for meta-analyses, systematic reviews and randomized controlled trials for the time range between 2011 and 2022. This search method produced 43 results, the exclusion and inclusion criteria are described in the following section.

### Inclusion/exclusion criteria and data synthesis

The included studies were selected using the following criteria. The search was directed towards studies investigating short-term and long-term effects for newborns whose cord was clamped early or delayed after birth, differentiated in two points of time. Only trials with mothers and newborns with > 37 weeks of gestational age were included. There were no restrictions regarding different birth modes. Studies were also included which investigated the impact of umbilical cord clamping on maternal factors to evaluate a potential disadvantage from cord clamping for the mother.

**Fig. 1:**
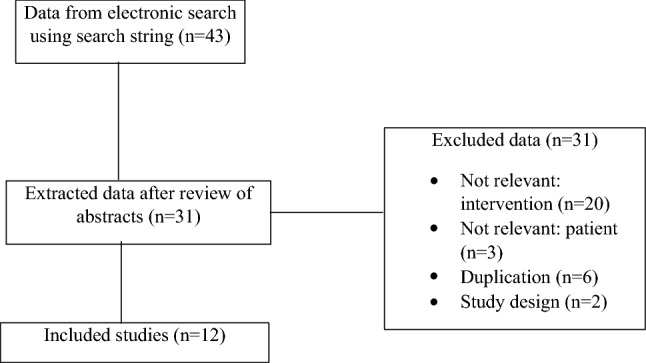
PRISMA flowchart

Trials examining other central interventions than umbilical cord clamping were excluded. The studies which showed effects for extremely low birthweight newborns or other preterm births before 37 weeks of gestation were also excluded. Two studies were excluded because of a protocol-based study design and a comment, which did not contain relevant information. The PRISMA flowchart (Fig. 1) shows the search procedure; the exact data from included and excluded studies are presented in the table for study characteristics.

### Quality of evidence

The quality of the systematic reviews and meta-analyses was evaluated by the AMSTAR 2 tool [12]. The conduct of RCTs is transparently presented using the CONSORT checklist to assess the risk of bias of the summary of results in this review [13].

## Results

### Study characteristics

The 12 included studies investigated the effects of different timing of umbilical cord clamping for newborns and mothers and the long-term effects for infants from 2 months to 3 years. The timing of umbilical cord clamping extended from immediately to 5 min or no pulsation of the umbilical cord, the literature review showed a high heterogeneity of management of cord clamping. Out of 12 articles, 3 were meta-analyses and 9 RCTs and no RCTs were included which were already included in one of the meta-analyses [14–25]. The study population in the different RCTs ranged from 56 to 720 participants [14, 15, 17, 19–24]. The details of included studies can be found in Table 1.

**Table 1:** Study characteristics

Study	Study design	Interventions/Setting/Country	Study population	Aims	Outcomes	Inclusion criteria
Mc Donald et al. (2013) [18]	Cochrane meta-analysis	Intervention groups: early cord clamping (within 15 s to ≤ 60 s) or late cord clamping (≥ 60 s to 3–5 min), different management of uterotonic medicine in both groups. Data from 1966 to 2012	15 randomized controlled trials, 3911 low-risk women	Maternal and neonatal effects of different timing of umbilical cord clamping	No significant risk for disadvantages for the mother; advantages for newborns from late cord clamping group, increased risk for phototherapy in late cord clamping group	Effects of early and late cord clamping for mothers and term infants
Salari et al. (2014) [23]	Prospective randomized clinical trial	Intervention groups: early cord clamping (< 10 s) or late cord clamping (≥ 3 min). Single-centre study in Iran over a 6-month period	56 low-risk women–newborn pairs delivered between 37 and 42 weeks of gestation	The effects of early or delayed cord clamping of normal-weight term infants on newborn haematocrit levels	Significant increase of haematocrit levels of newborns at 2 h and 18 h after birth. No difference in Apgar scores or duration of the third stage of labour	Effects of early and late cord clamping for low-risk mothers and term-newborns
Sun et al. (2017) [24]	Randomized controlled trial	Intervention groups: early cord clamping (< 60 s) or delayed cord clamping (> 60 s or after pulsation). Single-centre study in China	338 low-risk women and newborn pairs at or after 37 weeks gestational age	Maternal and neonatal effects of delayed umbilical cord clamping in caesarean section	Significant differences in residual blood in the placenta, postpartum haemorrhage, haemoglobin and haematocrit levels of heel blood (72 h after birth) and successful resuscitation	Effects of delayed cord clamping for mothers and term infants
Mercer et al. (2018) [19]	Randomized partially blinded controlled trial	Intervention groups: Early cord clamping (< 20 s) or delayed cord clamping (> 5 min) in vaginally delivered newborns. Cord milking (five times) instead of delayed cord clamping in caesarean sections. Single-centre study at a tertiary hospital in the USA	73 low-risk women and newborn pairs, singleton pregnancies and term labour	Effects of placental transfusion in brain myelination at 4 months of age. Short- and long-term effects after birth and at 48 h of life in haemoglobin, haematocrit and bilirubin levels	Significant increase in brain myelin in important areas for early life functional development and increase of ferritin levels in infants of 4 months of age	Long-term effects of early or delayed cord clamping for term infants
Chen et al. (2018) [15]	Randomized controlled trial	Intervention groups: immediate cord clamping (< 15 s) or delayed cord clamping (at 30, 60, 90, 120, 150, 180 s or no pulsation), the infant was held (10–15 cm) below the placenta	720 women–newborn pairs with low risks, labour between 37 + 0–41 + 6 weeks of gestation. Randomized into 8 groups (*n* = 90)	Evaluate effects and safety of different timing of umbilical cord clamping	Significant increase in haematocrit levels and no harmful effects for mother and newborn	Effects of delayed cord clamping, timing of cord clamping and disadvantageous effects for newborns and mothers
Purisch et al. (2019) [21]	Randomized clinical trial	Intervention groups: immediate cord clamping (15 s) or late cord clamping (60 s), measurements at two different hospitals (USA)	113 women, term singleton gestation (≥ 37 weeks)	Compare influence of maternal blood loss in scheduled caesarean delivery	No significant differences in maternal haemoglobin levels from preoperational to 1 day post-operational	Women with singleton pregnancies (≥ 37 weeks) and scheduled caesarean delivery, effects of cord clamping management for mothers
Zhao et al. (2019) [25]	Systematic review and meta-analysis	Intervention groups: early cord clamping (0–60 s) vs. late cord clamping (> 60 s or after pulsating). Data of long-term effects after neonatal period (preterm and term infants) published between 1960 and 2017	20 trails, 3733 infants Only randomized clinical trials	Long-term effects of delayed or early cord clamping on infants after neonatal period (preterm and term birth)	Significant advantages (haematological and iron status) from delayed cord clamping for term infants after 2–12 months	Effects of early and delayed cord clamping on term infants after newborn period, not preterm births
Rana et al. (2019) [22]	Randomized controlled trial	Intervention groups: early cord clamping (≤ 60 s) and late cord clamping (≥ 180 s). Single-centre clinical trial in November 2014 at a tertiary hospital in Nepal	332 low-risk mother–newborn pairs, term infants	Long-term effects of early vs. late cord clamping on infants at 12 months of age	Significant improvement of neurodevelopment at 12 months of age in the delayed cord clamping group (3 min), measured by the Ages and Stages Questionnaire. Significant increase of haemoglobin levels at 12 months	Effects of early or late cord clamping on infants
Li et al. (2020) [17]	Randomized controlled trial	Intervention groups: no labour analgesia and with labour analgesia (pain pump and pudendal nerve block by infiltration anaesthesia). Subgroups: early cord clamping (after mucus sucking) and delayed cord clamping (after pulsating). Subgroups were both performed in each intervention group. Single-centre study from China	288 mothers (age of 18–34 years) with perineal tear after delivery of a singleton term infant (37–41 weeks of gestation)	Effects of delayed cord clamping on pain during suturing perineal tears, the healing of the perineal wound and the maternal cooperation degree. Measuring was performed among verified pain scales	Significant difference in pain during suturing perineal tears advocating delayed cord clamping, more pain perception with labour analgesia was observed. Degree of cooperation (secondary outcome) differed, also advocating delayed cord clamping	Effects of early or delayed cord clamping on maternal pain perception after spontaneous labour of term infant
Fu et al. (2020) [16]	Systematic review and Meta-analysis	Intervention groups: early cord clamping (intermediate to ≤ 60 s) or delayed cord clamping (≥ 60– ≥ 180 s and no pulsation). Data from 1997–2017	13 (quasi) randomized controlled trials, 962–1982 infants for different effects. Singleton pregnancies and term infants	Effects of delayed cord clamping on haemoglobin, mean corpuscular volume and ferritin levels in infants of 2 months or older (max. 12 months)	Significant increase of haemoglobin, ferritin and MCV levels in delayed cord clamping groups for infants between 2 and 12 months of age	Long-term effects of early or delayed cord clamping for term infants
Ofojebe et al. (2021) [20]	Randomized controlled trial	Intervention groups: early cord clamping (0–15 s) and delayed cord clamping (60 s) at one hospital in Nigeria	102 low-risk newborn–mother pairs after spontaneous labour at 37–42 weeks of gestation	Mean haemoglobin and bilirubin levels of the newborn (at birth and 48 h after birth), maternal postpartum haemorrhage, infant anaemia and polycythaemia or need for phototherapy and proportion of respiratory symptoms	Significant advantages of delayed cord clamping and no significant maternal or neonatal complications	Effects of early or delayed cord clamping on term infants and mothers after spontaneous labour with low risks
Berg et al. (2021) [14]	Randomized controlled trial	Intervention groups: early cord clamping (≤ 60 s) or delayed cord clamping (≥ 180 s) at one tertiary hospital in Nepal	350 low-risk term infants, measurement with Ages and Stages Questionnaire at 3 years of age	Effects of early or delayed cord clamping on neurodevelopment at 3 years of age	No significant differences in ASQ scores accept the risk for affected gross motor development (girls) in the early cord clamping group	Effects of early or delayed cord clamping on term infants. In this study, infants from 34 weeks of gestational age were included, the mean gestational age was 39 + 0 ± 1 and 39 + 3 ± 1.1 which led to inclusion to this review

#### Neonatal outcomes

The results of 12 included studies show significant advantages in delayed cord clamping (different timings) for newborns and infants up to 12 months of age, as shown in Table 2. The advantages concern haemoglobin, haematocrit, iron and ferritin levels and mean corpuscular volumes for newborns and infants up to 12 months of age [16, 18, 19, 22–25]. In addition, delayed cord clamping seems to reduce the incidence of anaemia and iron deficiency anaemia in infants up to 12 months of age [16, 18, 22, 25]. Furthermore, the results show that delayed cord clamping seems to affect early neuronal development advantageously, measured by the Ages and Stages Questionnaire [26]. One meta-analysis showed jaundice requiring phototherapy for the delayed cord clamping group, another meta-analysis showed an increase in serum bilirubin for infants at 3–5 months of age [18, 25]. One result shows low haematocrit levels in the first hours after birth, but the confidence interval was large [18]. In summary, there are many advantages of delayed cord clamping and one possible disadvantage regarding the incidence of jaundice or need for phototherapy.

**Table 2:** Neonatal outcomes

Outcome	Number of participants	Statistical method	Effect size
APGAR score < 7 at 5 min [18]	1399	Risk ratio, 95% CI	1.23 [0.73, 2.07]
APGAR score at 1 min [24]	338	Mean difference (delayed/early)	9.52 ± 1.05/9.56 ± 1.08, *p* = 0.904
APGAR score at 5 min [24]	338	Mean difference (delayed/early)	9.84 ± 3.74/9.80 ± 0.50, *p* = 0.770
APGAR score at 5 min [23]	56	Mean difference (delayed/early)	9.3 ± 0.6/9.4 ± 0.6, *p* = 0.5
APGAR score at 1 min [19]	44	Median difference (delayed/early)	8(3–9)/8(2–9), *p* = 0.77
APGAR score at 5 min [19]	44	Median difference (delayed/early)	9(8–9)/9(5–9), *p* = 0.67
APGAR score at 1 min [21]	113	Median difference	0 (0, 0), *p* = 0.39
ARGAR score at 5 min [21]	113	Median difference	0 (0, 0), *p* = 0.26
Admission to SCN, NICU [18]	1675	Risk ratio, 95% CI	0.79 [0.48, 1.31]
Admission to NICU [21]	113	Risk difference	5.2 (− 2.2, 12.7) *p* = 0.36
Admission to neonatal department [15]	720	Mean difference	Not significant for each timing group (8 groups)
Respiratory distress [18]	835	Risk ratio, 95% CI	0.70 [0.22, 2.19]
Percentage of asphyxia resuscitation (successful) [24]	338	Count/Percentage (delayed/early)	12 (100%)/11 (55%), *p* = 0.016
Jaundice requiring phototherapy [18]	2324	Risk ratio, 95% CI	0.62 [0.41, 0.96]
Jaundice requiring phototherapy [21]	113	Risk difference	− 1.8 (− 5.3, 1.7) *p* = 0.50
Jaundice requiring phototherapy [20]	102	Risk ratio, 95% CI	[0.98, 1.04] *p* = 0.561
Jaundice requiring phototherapy[24]	338	Percentage (delayed vs. early)	11.8% vs. 12.4% *p* = 0.868
Jaundice requiring phototherapy [15]	720	Mean difference	Not significant for each timing-group (8 groups)
Clinical jaundice [18]	2098	Risk ratio, 95% CI	0.84 [0.66, 1.07]
Neonatal jaundice [20]	102	Risk ratio, 95% CI	1.0 [0.89, 1.15] *p* = 0.856
Serum bilirubin at 3–5 months [25]	169	Weighted MD, 95% CI	2.02 [1.59, 2.45] *p* < 0.00001
Mean infant bilirubin at birth (g/dL) [20]	102	Mean difference, 95% CI	− 0.04 [ − 0.38, 0.30] *p* = 0.815
Mean infant bilirubin after 48 h (g/dL) [20]	102	Mean difference, 95% CI	− 0.17 [ − 0.55, 0.21] *p* = 0.380
Hyperbilirubinemia (TC-measurement) [24]	338	Percentage (delayed vs. early)	14.8% vs. 14.2% *p* = 0.877
Highest bilirubin (mmol/L) [24]	338	Mean difference (delayed/early)	10.599 ± 1.885 / 10.374 ± 1.776, *p* = 0.260
Bilirubin (mg/dL) at 72 h [15]	720	Mean difference ± SD	Not significant for each timing group (8 groups)
Bilirubin > 12.9 mg/dL at 72 h [15]	720	Mean difference	Not significant for each timing group (8 groups)
BilliTool, high-risk zone (billitool.org) [19]	44	Median difference (delayed/early)	2(9)/2(10), *p* = >0.99
Peak total bilirubin (mg/dL) [19]	44	Mean difference (delayed/early)	8.5 ± 4/9.1 ± 2, *p* = 0.56
Polycythaemia [18]	1025	Risk ratio, 95% CI	0.39 [0.12, 1.27]
Polycythaemia (haematocrit > 65%) [20]	102	Risk ratio, 95% CI	0.0, undefined
Cord haemoglobin (g/dL) [18]	696	Mean difference, 95% CI	0.41 [0.15, 0.66]
Cord haemoglobin (g/L) [24]	338	Mean difference (delayed vs. early)	150.633 ± 11.037/149.964 ± 10.766, *p* = 0.564
Mean cord haemoglobin at birth (g/dL) [20]	102	Mean difference, 95% CI	− 0.40 [0.29, 0.51] *p* < 0.001
Newborn haemoglobin (g/dL) [18]	671	Mean difference, 95% CI	− 2.17 [− 4.06, − 0.28]
Newborn haemoglobin (g/dl) at 2 h [23]	56	Mean difference (delayed vs. early)	17.2 ± 2/15.7 ± 1.6, *p* = 0.004
Newborn haemoglobin (g/dl) at 18 h [23]	56	Mean difference (delayed vs. early)	18.7 ± 1.7/16.7 ± 2, *p* = 0.0002
Newborn haemoglobin (g/L) at 72 h (heel blood) [24]	338	Mean difference (delayed vs. early)	188.520 ± 14.292/171.733 ± 10.809, *p* = 0.0001
Newborn haemoglobin (g/dL) at 24–48 h [18]	884	Mean difference, 95% CI	− 1.49 [ − 1.78, − 1.21]
Newborn haemoglobin (g/dL) at 24–72 h [21]	90	Mean difference, 95% CI	1.67 [0.75, 2.59] *p* < 0.001
Mean newborn haemoglobin (g/dL) at 48 h [20]	102	Mean difference, 95% CI	− 1.35 [0.80, 1.90] *p* < 0.001
Newborn haemoglobin (g/dL) at 48 h [19]	44	Mean difference (delayed vs. early)	19.1 ± 2/18.0 ± 2, *p* = 0.06
Infant haemoglobin (g/dL) at 3–6 months [18]	1115	Mean difference, 95% CI	− 0.15 [ − 0.48, 0.19]
Infant haemoglobin (g/dL) at 4 months [19]	44	Mean difference (delayed vs. early)	11.7 ± 1.0/11.7 ± 0.7, *p* = 0.93
Infant haemoglobin (g/dL) ≥ 6 months [25]	1670	Mean difference, 95% CI	0.15 [0.06, 0.25] *p* = 0.002
Infant haemoglobin (g/dL) 2–12 months [16]	1982	Mean difference, 95% CI	0.4678 [0.1515, 0.7841] *p* = 0.004
Infant haemoglobin (g/dL) at 12 months [22]	326	MLR (B), 95% CI	1.8 [0.6, 3.1], *p* = 0.004
Low Infant haemoglobin (g/dL) at 3–6 months [18]	954	Risk ratio, 95% CI	1.05 [0.79, 1.39]
Cord haematocrit (%) [24]	338	Mean difference (delayed vs. early)	45.199 ± 3.509/45.534 ± 4.226, *p* = 0.482
Cord haematocrit (%) [19]	44	Mean difference (delayed vs. early)	43.7 ± 6/45.8 ± 5, *p* = 0.25
Newborn haematocrit (%) at 2 h [23]	56	Mean difference (delayed vs. early)	49.5 ± 4.4/45.1 ± 4, *p* = 0.0003
Newborn haematocrit (%) at 18 h [23]	56	Mean difference (delayed vs. early)	52.9 ± 4.3/47.7 ± 5.5, *p* = 0.0002
Newborn haematocrit (%) at 24 h [18]	180	Mean difference, 95% CI	− 4.40 [ − 5.71, − 3.09]
Newborn haematocrit (%) at 48 h [19]	44	Mean difference (delayed vs. early)	57.6 ± 6/53.1 ± 6, *p* = 0.01
Newborn haematocrit (%) at 72 h (heel blood) [24]	338	Mean difference (delayed vs. early)	51.614 ± 6.174/45.139 ± 4.306, *p* = <0.0001
Infant haematocrit at 3–5 months [18]	160	Mean difference, 95% CI	− 0.40 [ − 1.48, 0.68]
Infant haematocrit (%) at 4 months [19]	44	Mean difference (delayed/early)	34 ± 2.3/34 ± 2.4, *p* = 0.76
Haematocrit at 24 h (%) [15]:			
Cord clamping < 15 s	90	Mean difference ± SD	56.5 ± 6.4, *p* < 0.001
Cord clamping at 30 s	90	Mean difference ± SD	57.3 ± 6.5, *p* < 0.001
Cord clamping at 60 s	90	Mean difference ± SD	58.8 ± 5.9, *p* < 0.001
Cord clamping at 90 s	90	Mean difference ± SD	59.7 ± 8.7, *p* < 0.001
Cord clamping at 120 s	90	Mean difference ± SD	59.5 ± 6.6, *p* < 0.001
Cord clamping at 150 s	90	Mean difference ± SD	59.7 ± 6.8, *p* < 0.001
Cord clamping at 180 s	90	Mean difference ± SD	60.3 ± 5.4, *p* < 0.001
Cord clamping “no pulsation”	90	Mean difference ± SD	61.0 ± 6.0, *p* < 0.001
Low infant haematocrit at 6 h (< 45%) [18]	272	Risk ratio, 95% CI	16.18 [2.05, 127.37]
Low infant haematocrit at 24–48 h (< 45%) [18]	268	Risk ratio, 95% CI	6.03 [2.27, 16.07]
Low infant haematocrit at birth-48 h (anaemia < 45%) [20]	102	Risk ratio, 95% CI	0.0, undefined
Anaemia incidence (< 45%) [24]	56	Percentage (delayed vs. early)	3.7%/31%, *p* = 0.008
Infant iron deficiency at 3–6 months [18]	1152	Risk ratio, 95% CI	2.65 [1.04, 6.73]
Iron deficiency < 6 months [25]	507	Risk ratio, 95% CI	0.13 [0.04, 0.44] *p* = 0.0009
≥ 6 months [25]	1071	Risk ratio, 95% CI	0.55 [0.43, 0.72] *p* < 0.00001
Birthweight (g) [18]	3139	Mean difference, 95% CI	− 101.18 [ − 157.59, − 44.76]
Birthweight (g) [21]	113	Mean difference, 95% CI	− 43 (− 195, 109) *p* = 0.57
Not breastfeeding at one month [18]	268	Risk ratio, 95%	1.10 [1.00, 1.20]
Not breastfeeding at discharge and 2–6 months later [18]		Risk ratio, 95%	Not significant
Neurodevelopment at 4 months (ASQ problem-solving score) [18]	365	Mean difference, 95% CI	− 1.80 [ − 3.38, − 0.22] Not significant
Further ASQ questions and total score (4 months) [18]	365	Risk Ratio, 95% CI	0.43 [0.26, 0.71], *p* < 0.001 NNT 11 (7–35)
Neurodevelopment at 12 months (ASQ total score) [22]	332	Mean difference, 95% CI	4.4 [1.8, 6.9], *p* = 0.001
Neurodevelopment at 12 months (ASQ total score) [22]	283	Risk Ratio, 95% CI	0.48 [0.28, 0.79], *p* = 0.003, NNT 11 (7–34)
ASQ: Communication (12 months) [22]	332	Mean difference, 95% CI	0.8 [0.2, 1.3], *p* = 0.008
ASQ: Communication (12 months) [22]	283	Risk Ratio, 95% CI	0.61 [0.39, 0.95], *p* = 0.03, NNT 14 (8–141)
ASQ: Gross motor (12 months) [22]	332	Risk Ratio, 95% CI	0.54 [0.34, 0.83], *p* = 0.004
ASQ: Personal-social (12 months) [22]	332	Mean difference, 95% CI	1.5 [0.7, 2.3], *p* < 0.001
ASQ: Personal-social (12 months) [22]	283	Risk Ratio, 95% CI	Not significant
ASQ: Fine motor, problem solving (12 months) [22]	332	Mean difference, 95% CI	Not significant
ASQ: Total score, all parameters at 3 years [14]	350	Percentage (delayed vs. early)	6 (6.3%) vs. 14 (18.9%), *p* = 0.02
ASQ: Gross motor (girls) at 3 years, delayed development [14]	350		
Symptoms of infection during first 4 months [18] Fever, diarrhoea, loose stools, hard stools, abdominal pain, vomiting, cough, breathing difficulties, rhinorrhoea, nasal congestion, rash, crying, tiredness, visit paediatrician/other doctor, antibiotics, admitted to hospital	360	Risk ratio, 95% CI	Not significant
Respiratory symptoms [20]	102		0.0, undefined
Neonatal crying/breathing established before cord clamping [21]	78	Risk difference, 95% CI	46.4 [31.7, 61.1] *p* < 0.001
Placental weight(g) [21]	113	Mean difference, 95% CI	− 38 [− 81, 6] *p* = 0.09
Residue blood (ml) (Placenta) [24]	338	Mean difference (delayed/early)	46.278 ± 39.205/95.301 ± 66.954, *p* = < 0.0001
Neonatal temperature (°C) [21]	113	Median difference	0 (− 0.1, –0.1) *p* = 0.33
Umbilical cord measures [21] Arterial base excess Cord venous/ arterial pH, venous base excess	105–109	Median difference	− 1.1 (− 2.3, –0.1) *p* = 0.004
Umbilical cord haemoglobin g/dL (venous) [21]	113	Mean difference, 95% CI	0.07 [− 0.42, 0.56] *p* = 0.78
Incidence of anaemia ≥ 6 months [25]	1717	Risk ratio, 95% CI	0.92 [0.87, 0.99] *p* = 0.02
Iron deficiency anaemia 4–12 months [25]	1799	Risk ratio, 95% CI	0.68 [0.49, 0.94] *p* = 0.02
Mean corpuscular volume (fL) at 4 months	44	Mean difference (delayed/early)	81.4 ± 4.0/81.5 ± 3.7, *p* = 0.94
Mean corpuscular volume < 6 months [25]	661	Mean difference, 95% CI	0.33 [0.15, 0.51] *p* = 0.0003
Mean corpuscular volume at 2–12 months [16]	962	Mean difference, 95% CI	0.5751 [0.1637, 0.9865] *p* = 0.006
Serum iron at 2–4 months [25]	570	Mean difference, 95% CI	0.23 [0.06, 0.40] n *p* = 0.007
Total body iron at 4–6 months [25]	578	Mean difference, 95% CI	0.45 [0.29, 0.62] *p* < 0.00001
Body iron at 6 months [25]	235	Weighted MD, 95% CI	20.80 [6.39, 35.13] *p* = 0.01
Stored iron at 6 months [25]	235	Weighted MD, 95% CI	19.90 [7.67, 32.12] *p* = 0.0001
Cord ferritin ng/dL [19]	44	Mean difference (delayed/early)	145 ± 92/141 ± 93, *p* = 0.89
Serum ferritin < 6 months [25]	975	Mean difference, 95% CI	1.22 [0.47, 1.98] *p* = 0.01
≥ 6 months [25]	1867	Mean difference, 95% CI	2.37 [0.99, 3.76] *p* = 0.0008
Serum ferritin at 2–12 months [16]	1956	Mean difference, 95% CI	2.1450 [1.0431, 3.2470] *p* = 0.0001
Ferritin (ng/dL) at 4 months [19]	44	Mean difference (delayed/early)	96.4 ± 58/65.3 ± 32, *p* = 0.03
Log serum-ferritin at 4 months [19]	44	Mean difference (delayed/early)	4.4 ± 0.5/4.1 ± 0.5, *p* = 0.03
Ferritin at 12 months [22]	326	MLR (B), 95% CI	0.09 [ − 0.5, 6.3], *p* = 0.09
Transferrin saturation at 2–12 months [25]	874	Mean difference, 95% CI	1.05 [0.53, 1.57] *p* < 0.0001
Transferrin (mg/dL) at 4 months [19]	44	Mean difference (delayed/early)	228 ± 31/239 ± 35, *p* = 0.28
Soluble transferrin receptor (mg/L) at 4 months [19]	44	Mean difference (delayed/early)	3.8 ± 0.9/3.8 ± 0.8, *p* = 0.93
Reticulocyte haemoglobin at 4 months [25]	343	Weighted MD, 95% CI	0.70 [0.28, 1.12] *p* = 0.001
Reticulocyte count at 4 months [25]	343		3.00 [0.67, 5.33] *p* = 0.01
Comparison of myelin content (measurement with MRI, Voxel-wise VFm) at 4 months [19]	44	General linear model, unpaired t-test and permutation testing	Colour-scale: *p* = 0.05 for several brain areas
Dichotomous comparison of myelin content and ferritin (measurement with MRI, Voxel-wise VFm) at 4 months [19]	44		Colour-scale: *p* = 0.05 to 0.01 for several brain areas

### Maternal outcomes

Table 3 shows the results of seven included studies which investigated effects for the mother of different timing of umbilical cord clamping. There seems to be no disadvantages for mothers when the cord is clamped after a delay. Sun et al. stated a significant reduction in blood loss after delayed cord clamping, which indicates a potential advantage for the mother [24]. One trial showed a beneficial effect on pain during suturing of perineal tears measured by different scales (Numeric Rating Scale, Visual Analogue Scale, Verbal Rating Scale, Faces Pain Scale) from late cord clamping with different methods of labour analgesia [17]. However, it has to be critically evaluated if this effect shows a correlation with the timing of umbilical cord clamping. In summary, it can be assumed that delayed cord clamping is safe for the mother, even though there were differences in management of uterotonics used for the third stage of labour.

**Table 3:** Maternal outcomes

Outcome	Number of participants	Statistical method	Effect size
Severe postpartum haemorrhage > 1000 ml [18]	2066	Risk ratio, 95% CI	1.04 [0.65, 1.65]
Severe postpartum haemorrhage > 1000 ml [21]	113	Risk difference	1.7 (− 9.5, 12.9) *p* > 0.99
Severe postpartum haemorrhage > 1000 ml [15]	720	Mean difference ± SD	Not significant for each timing group (8 groups)
Postpartum haemorrhage (ml) [24]	338	Mean difference (delayed/early)	156.775 / 221.627, *p* = <0.0001
Postpartum haemorrhage > 500 ml [18]	2260	Risk ratio, 95% CI	1.17 [0.94, 1.44]
Postpartum blood loss > 500 ml [15]	720	Mean difference ± SD	Not significant for each timing group (8 groups)
Mean maternal blood loss ≥ 500 ml [20]	102	Risk ratio, 95% CI	0.6 [0.26, 0.79] *p* = 0.653
Mean blood loss [18]	1345	Mean difference, 95% CI	5.11 [ − 23.18, 33.39]
Mean blood loss (ml)	720	Mean difference ± SD	Not significant for each timing-group (8 groups)
Estimated blood loss [21]	113	Median difference, 95% CI	0 [0, 0] *p* = 0.13
Maternal haemoglobin (g/dl) 24 to 72 h postpartum [18]	1128	Mean difference, 95% CI	− 0.12 [ − 0.30, 0.06]
Maternal haemoglobin (g/dl) 1 day post-operational [21]	113	Mean difference, 95% CI	0.12 g/dL [− 0.22 to 0.46]
Need for blood transfusion [18]	1345	Risk ratio, 95% CI	1.02 [0.44, 2.37]
Need for blood transfusion [21]	113	Risk difference, 95% CI	− 3.6 [− 8.4, 1.3] *p* = 0.24
Need for manual removal of placenta [18]	1515	Risk ratio, 95% CI	1.59 [0.78, 3.26]
Length of third stage > 30 min [18]	1345	Risk ratio, 95% CI	1.18 [0.55, 2.52]
Length of third stage > 60 min [18]	1345	Risk ratio, 95% CI	1.11 [0.33, 3.74]
Duration of third stage (minutes) [23]	56	Mean difference (delayed/early)	8.9 ± 5/10.2 ± 3.7, *p* = 0.2
Duration of third stage (minutes) [15]	720	Mean difference ± SD	Not significant for each timing group (8 groups)
Need for therapeutic uterotonics [18]	963	Risk ratio, 95% CI	0.94 [0.74, 1.20]
Uterotonic administration [21]	113	Risk difference	− 0.13 (− 9.33, 9.56) *p* > 0.99
Total surgical time [21]	113	Median difference, 95% CI	3.0 [− 6.0, 12.0] *p* = 0.18
Hysterectomy [21]	113	Risk difference	0.1 (− 4.8, 4.9) *p* > 0.99
Pain during suturing perineal tears [17]	288	Mean value of pain scores (NRS, VAS, VRS, FPS) (Mann–Whitney-*U* test)	NRS: *p* < 0.001 VAS: *p* < 0.001; VRS: *p* < 0.001 FPS: *p* < 0.001
With labour analgesia (Delayed vs. early cord clamping) [17]	123		
No labour analgesia (Delayed vs. early cord clamping) [17]	165		NRS: *p* < 0.001; VAS: *p* < 0.001; VRS: *p* < 0.001; FPS: *p* < 0.001
Delayed cord clamping (no labour analgesia vs. labour analgesia) [17]	147		NRS: *p* = 0.007; VAS: *p* = 0.29; VRS: *p* = 0.005; FPS: *p* = 0.005
Early cord clamping (no labour analgesia vs. labour analgesia) [17]	141		BRS: *p* = 0.685; VAS: *p* = 0.418; VRS: *p* = 0.005; FPS: *p* = 0.053
Degree of cooperation during suturing perineal tears [17]	165	Cooperation rate (%) (Chi-square test)	78.57% vs. 29.63% (*x*^2^ = 39.839) *p* < 0.001
Delayed cord clamping vs. early cord clamping (no analgesia) [17]			90.48% vs. 45% (*x*^2^ = 29.351) *p* < 0.001
Delayed cord clamping vs. early cord clamping (with analgesia) [17]	123		
Delayed cord clamping with analgesia vs. without analgesia [17]			
Early cord clamping with analgesia vs. without analgesia [17]	288		Not significant

### Quality of evidence

Overall, the quality of all included studies, RCTs and meta-analyses seems to be moderate or high. Table 4 shows the results of the evidence evaluation of the meta-analyses via AMSTAR-2 score and Table 5 shows the results of the evidence evaluation of the RCTs via CONSORT.

**Table 4:** Quality of evidence, AMSTAR-2-Score

Dimensions of AMSTAR-2	Mc Donald et al. (2013) [18]	Zhao et al. (2019) [25]	Fu et al. (2020) [16]
1. Did the research questions and inclusion criteria for the review include the components of PICO?	Yes	Yes	Yes
2. Did the report of the review contain an explicit statement that the review methods were established prior to the conduct of the review and did the report justify any significant deviations from the protocol?	Yes	Yes	Yes
3. Did the review authors explain their selection of the study designs for inclusion in the review?	Yes	Yes	Yes
4. Did the review authors use a comprehensive literature search strategy?	Yes	Partial yes	Partial yes
5. Did the review authors perform study selection in duplicate?	Yes	Yes	Yes
6. Did the review authors perform data extraction in duplicate?	Yes	Yes	Yes
7. Did the review authors provide a list of excluded studies and justify the exclusions?	Yes	No	Partial yes
8. Did the review authors describe the included studies in adequate detail?	Yes	Yes	Yes
9. Did the review authors use a satisfactory technique for assessing the risk of bias (RoB) in individual studies that were included in the review?	Yes	Yes	No
10. Did the review authors report on the sources of funding for the studies included in the review?	Yes	No	Yes
11. If meta-analysis was performed, did the review authors use appropriate methods for statistical combination of results?	Yes	Yes	Yes
12. If meta-analysis was performed, did the review authors assess the potential impact of RoB in individual studies on the results of the meta-analysis or other evidence synthesis?	Yes	Yes	No
13. Did the review authors account for RoB in individual studies when interpreting/ discussing the results of the review?	Yes	Yes	No
14. Did the review authors provide a satisfactory explanation for and discussion of any heterogeneity observed in the results of the review?	Yes	Yes	Yes
15. If they performed quantitative synthesis, did the review authors carry out an adequate investigation of publication bias (small study bias) and discuss its likely impact on the results of the review?	Yes	Yes	Yes
16. Did the review authors report any potential sources of conflict of interest, including any funding they received for conducting the review?	Yes	Yes	Yes

**Table 5:** Quality of evidence, CONSORT

Dimensions of CONSORT	Salari et al. (2014) [13, 23]	Sun et al. (2017) [13, 24]	Chen et al. (2018) [13, 15]	Mercer et al. (2018) [13, 19]	Purish et al. (2019) [13, 21]	Rana et al. (2019) [13, 22]	Li et al. (2020) [13, 17]	Berg et al. (2021) [13, 14]	Ofrojebe et al. (2021) [13, 20]
1a. Identification as a randomized trial in the title	Yes	No	Yes	Yes	Yes	Yes	Yes	Yes	Yes
1b. Structured summary of trial design, methods, results, and conclusions (for specific guidance see CONSORT for abstracts)	Yes	Yes	Yes	Yes	Yes	Yes	Yes	Yes	Yes
2a. Scientific background and explanation of rationale	Yes	Yes	Yes	Yes	Yes	Yes	Yes	Yes	Yes
2b. Specific objectives or hypotheses	Yes	Yes	Yes	Yes	Yes	Yes	Yes	Yes	Yes
3a. Description of trial design (such as parallel, factorial) including allocation ratio	Yes	Yes	Yes	Yes	Yes	Yes		Yes	Yes
3b. Important changes to methods after trial commencement (such as eligibility criteria), with reasons	Yes	No	No	No	No	No	No	No	No
4a. Eligibility criteria for participants	Yes	Yes	Yes	Yes	Yes	Yes	Yes	Yes	Yes
4b. Settings and locations where the data were collected	Yes	Yes	Yes	Yes	Yes	Yes	Yes	Yes	Yes
5. The interventions for each group with sufficient details to allow replication, including how and when they were actually administered	Yes	Yes	Yes	Yes	Yes	Yes	Yes	Yes	Yes
6a. Completely defined pre-specified primary and secondary outcome measures, including how and when they were assessed	Yes	Yes	Yes	Yes	Yes	Yes	Yes	Yes	Yes
6b. Any changes to trial outcomes after the trial commenced, with reasons	Yes	Yes	No	No	No	No	No	No	No
7a. How sample size was determined	Yes	No	Yes	Yes	Yes	Yes	No	Yes	Yes
7b. When applicable, explanation of any interim analyses and stopping guidelines	No	Yes	No	No	No	No	No	Yes	No
8a. Method used to generate the random allocation sequence	Yes	No	Yes	Yes	Yes	Yes	Yes	Yes	Yes
8b. Type of randomization; details of any restriction (such as blocking and block size)	Yes	No	Yes	Yes	Yes	Yes	Yes	Yes	Yes
9. Mechanism used to implement the random allocation sequence (such as sequentially numbered containers), describing any steps taken to conceal the sequence until interventions were assigned	Yes	No	Yes	Yes	Yes	Yes	Yes	Yes	Yes
10. Who generated the random allocation sequence, who enrolled participants, and who assigned participants to interventions	Yes	No	Yes	Yes	Yes	Yes	Yes	Yes	Yes
11a. If done, who was blinded after assignment to interventions (for example, participants, care providers, those assessing outcomes) and how	Yes	No	Yes	Yes	Yes	Yes	Yes	Yes	Yes
11b. If relevant, description of the similarity of interventions	Yes	Yes	Yes	Yes	Yes	Yes	Yes	Yes	Yes
12a. Statistical methods used to compare groups for primary and secondary outcomes	Yes	Yes	Yes	Yes	Yes	Yes	Yes	Yes	Yes
12b. Methods for additional analyses, such as subgroup analyses and adjusted analyses	No	No	No	Yes	No	Yes	Yes	Yes	Yes
13a. For each group, the numbers of participants who were randomly assigned, received intended treatment, and were analyzed for the primary outcome	Yes	Yes	Yes	Yes	Yes	Yes	Yes	Yes	Yes
13b. For each group, losses and exclusions after randomization, together with reasons	No	No	No	No	No	Yes	Yes	Yes	No
14a. Dates defining the periods of recruitment and follow-up	Yes	No	Yes	Yes	Yes	Yes	Yes	Yes	Yes
14b. Why the trial ended or was stopped	No	No	No	No	No	No	No	No	No
15. A table showing baseline demographic and clinical characteristics for each group			Yes	Yes	Yes	Yes	Yes	Yes	Yes
16. For each group, number of participants (denominator) included in each analysis and whether the analysis was by original assigned groups	Yes	No	Yes	Yes	Yes	Yes	Yes	Yes	Yes
17a. For each primary and secondary outcome, results for each group, and the estimated effect size and its precision (such as 95% confidence interval)	Yes	Yes	Yes	Yes	Yes	Yes	Yes	Yes	Yes
17b. For binary outcomes, presentation of both absolute and relative effect sizes is recommended	No	No	Yes	No	Yes	No	Yes	No	Yes
18. Results of any other analyses performed, including subgroup analyses and adjusted analyses, distinguishing pre-specified from exploratory	No	No	No	Yes	No	Yes	Yes	Yes	No
19. All important harms or unintended effects in each group (for specific guidance see CONSORT for harms)	No	Yes	No	No	Yes	No	No	Yes	No
20. Trial limitations, addressing sources of potential bias, imprecision, and, if relevant, multiplicity of analyses	No	No	Yes	No	Yes	Yes	Yes	Yes	Yes
21. Generalisability (external validity, applicability) of the trial findings	Yes	Yes	Yes	Yes	Yes	Yes	Yes	Yes	Yes
22. Interpretation consistent with results, balancing benefits and harms, and considering other relevant evidence	Yes	Yes	Yes	Yes	Yes	Yes	Yes	Yes	Yes
23. Registration number and name of trial registry	No	No	No	Yes	Yes	No	Yes	Yes	Yes
24. Where the full trial protocol can be accessed, if available	Yes	Yes	Yes	Yes	Yes	Yes	Yes	Yes	Yes
25. Sources of funding and other support (such as supply of drugs), role of funders	Yes	No	Yes	Yes	Yes	Yes	Yes	Yes	Yes

There is a medium–high to high quality of the included meta-analyses (11 of 16 [16], 13 of 16 [25], 16 of 16 [18], respectively, which fulfilled criteria according to AMSTAR-2).

Among the included RCTs, 5 studies showed high quality (30–33 of 37 CONCORT criteria met) [13, 14] and 3 studies showed medium–high quality (28 and 29 of 37 CONSORT criteria met, respectively) [13, 15], whereas 1 study was of insufficient quality or could only be inadequately assessed via CONSORT (19 criteria met) [13, 24].

## Discussion

### Results’ overview

The aim of this review was to evaluate the timing of umbilical cord clamping for term infants from 37 + 0 weeks gestational age, to describe the effects of the timing of cord clamping for newborns and mothers and to improve the evidence-based work of midwives in Germany. The results of this review regarding the timing of umbilical cord clamping arose from low-risk populations in most of the trials [14, 15, 18–20, 22–25]. The majority of infants were born vaginally, three of the included trials also included primary caesarean sections [18, 21, 24]. Furthermore, most of the included mother–newborn pairs had singleton pregnancies [15–17, 20, 21, 24]. The results may not apply to vaginal-operative deliveries or other birth risks; however, overall, there were no birth risks such as asphyxia, placental anomalies, intrauterine growth restrictions, differences in APGAR scores between groups or differences in neonatal mortality and morbidity [17, 18, 21–25].

The evaluation about the exact timing of umbilical cord clamping in term infants cannot be concluded, the included trials report about many advantages for newborn and infants up to 12 months of age from delayed cord clamping, but all the included trials reached this outcome for different timings of cord clamping. The timing of early cord clamping ranged from immediately to < 60 s, the timing of delayed cord clamping ranged from 60 s after birth up to cessation of umbilical cord pulsation, which is a broad description because of the individual, physiological differences depending on the time of onset of respiration. However, delayed cord clamping > 60 s seems to be advantageous for newborns in terms of iron stores and its short and long-term effect up to 12 months of age [16, 18–21, 23–25]. Timing of cord clamping in term infants could have an impact on neuronal development [14, 18, 22]. Some trials reported an increase in bilirubin levels or clinical jaundice which increases the need for phototherapy, but other risk factors were not strictly considered [18, 25]. Delayed cord clamping for different timings seems to have no disadvantages for mothers; one trial described pain reduction while suturing perineal tears, but this result can also be correlated with psychological satisfaction with the birth [17].

There is need for further research to evaluate if there are different results in terms of advantageous effects for newborns when the mother’s haemoglobin is low at the start of labour. One trial measured the effects of change of mother’s haemoglobin from early or delayed cord clamping which was not significant, but did not measure the correlation between the strength of effects for newborns and their mother’s haemoglobin [21]. There is also heterogeneity in the definition of delayed cord clamping. Maybe the measurement of effects of placental blood perfusion after birth should include the physiological process of adaptation. What the duration of umbilical cord pulsation depends on should also be evaluated, and whether a physiological time of cord clamping can be determined.

According to the actual AWMF guideline for vaginal birth at term, the results for timing of umbilical cord clamping are equivalent. They recommend waiting at least 1 min up to 5 min before cord clamping or to wait until the cord stops pulsating, depending on whether active or passive management of the third stage of labour is chosen [9]. Regarding the present research question, the authors of the AWMF guideline also found no disadvantageous effect for the mother and advantageous effects for newborn and infants up to 4 months of age from delayed cord clamping after 1 min [9]. It should be noted that this review did not include the placement of the newborn while waiting for cord clamping after a vaginal delivery. This is due to the fact that the usual management directly after birth and the actual recommendations emphasize skin-to-skin contact and only the minimum of intervention in this “sensitive phase” [9]. This recommendation is also given by the paediatric guidelines for term newborns after vaginal birth, i.e. skin-to-skin contact should be enabled before cord clamping [10]. They also point out that physiological processes for the decision of the timing of cord clamping should be observed, and the adaptation of the cardiovascular system and respiration is decisive for the health of the newborn [10]. The recommendations of the World Health Organization also include the definition of delayed cord clamping is > 1 min up to 3 min, and point out that there has to be research to evaluate a physiological timing of cord clamping [27].

### Limitations and risk of bias

The inclusion criteria were strictly observed and evaluated if the trial was appropriate (Table 1). A risk of selection bias could be present, as only one person assessed the inclusion process. However, the inclusion process took place using the PICO pattern to make sure the research questions and aims are matching. Despite the orientation on systematic search by creating a search string, there is a risk of not accessing all relevant articles, especially because of language restrictions (German and English). The data extraction and synthesis were also made by one person, but reviewed by an independent researcher; however, this could have led to an observer bias. The data collected from all included studies are shown in Tables 2 and 3. The structure for data extraction was to collect all relevant data, primary and secondary outcomes independent of the significance, to eliminate reporting bias. The data synthesis consciously produced a sort of performance bias because the aim was to evaluate the timing of umbilical cord clamping, and nearly every included study had a different timing of cord clamping. It is unavoidable that there is a risk of bias for the search strategy because the search was not conducted in many databases and maybe could not include every trial concerning the effects of umbilical cord clamping.

Tables 4 and 5 show the methodological quality of each included trial or meta-analysis. Nevertheless, all the biases created in the included trials lead to an increased risk of bias in this review. Some of the included RCTs did not perform a structured randomization, and the blinding of patients or research staff was not completely described in every RCT. The determination of cord clamping by stopwatch was performed in many trials, and some did not describe in detail how the timing was measured. As mentioned, the placement of the newborn above or below the placenta and the impact of gravity were not considered in this review, some studies mentioned placement and others did not, and this could have an impact on the effects from cord clamping.

In summary, there is a risk of different biases and a limitation in informative value; however, the results of this review correspond to the actual recommendations for practitioners in Germany, and the review gives an important impulse for further research to evaluate the exact timing of umbilical cord clamping, the effects of waiting until pulsation has stopped and also to explore the boundaries of waiting 1 min before cord clamping.

### Authors’ conclusion

This narrative review shows that delayed cord clamping on term infants > 37 weeks of gestational age, with no or low birth risks, born vaginally or by primary caesarean section, has advantageous effects for newborns and infants up to 12 months of age. This management of umbilical cord clamping could reduce the incidence of anaemia and seems to correlate with a better neurodevelopment during the early life of infants. In addition, it shows that there are no adverse effects for the mothers, so the management of delayed cord clamping seems to be safe concerning postpartum haemorrhage and high blood loss. Unfortunately, the second part of this central research question about the exact timing of umbilical cord clamping leading to the aforementioned advantages cannot be answered. The critical value for both early and delayed cord clamping has to be determined in further research to produce exact results for their implementation into practice. Rana et al. showed a cut-off point of 61 s for early cord clamping, other authors describe advantageous effects from 60 to 120 s, while the effects could be stronger when the umbilical cord was cut later because of the perfusion of placental blood [22]. In contrast, Chen et al. showed no significant increase in haematocrit levels in newborns after 90 s [15]. Further research should address the question of if there are any signs to improve the knowledge about physiological umbilical cord clamping to achieve the advantages of longer placental perfusion for each individual term infant.

## Data Availability

Data on the research, evaluation and assessment of the studies may be available upon request. Please contact the corresponding author.

## Abbreviations

AMSTAR 2: Assessment of multiple systematic reviews, V2
APGAR: Atmung, Puls, Grundtonus, Aussehen, Reflexe (skin colour, pulse rate, reflex irritability grimace, muscle tone, respiratory effort)
AWMF: Arbeitsgemeinschaft der Wissenschaftlichen Medizinischen Fachgesellschaften e. V (Working Group of Scientific Medical Societies)
BRS: Brief Resilience Scale
CI: Confidence interval
FPS: Faces Pain Scale
NRS: Numeric Pain Rating Scale
PICO: Patient-Intervention-Control-Outcome
PRISMA: Preferred Reporting Items for Systematic Review and Meta-Analysis
RCT: Randomized Controlled Trial
SD: Standard deviation
UK: United Kingdom
VAS: Visual Analogue Scale
VRS: Verbal Pain Rating Scale
WHO: World Health Organization
